# Mendelian Susceptibility to Mycobacterial Diseases (MSMD) in a 13-Year-Old Ethiopian Girl with Autosomal Dominant Interferon Gamma Receptor 1(IFN-*γ* R1) Defect: A Clinical Diagnostic and Treatment Challenge

**DOI:** 10.1155/2022/6534009

**Published:** 2022-09-23

**Authors:** Netsanet Azene Gebeyehu, Solomie Jebessa Deribessa, Freeman Alexandra, Messay Tesfaye Demissie, W Mihretu Gebre, Aklilu Melaku Gebremariam, Dagmawit Mitiku Engliz, Tizita Yosef Kidane, Lidya Million Bekele, Abate Yeshidinber Weldetsadik

**Affiliations:** ^1^Department of Dermatology and Venereology, Addis Ababa University College of Health Sciences, Addis Ababa, Ethiopia; ^2^Pediatrics Infectious Disease Specialist, Department of Pediatrics and Child Health, St.Paul's Hospital Millennium Medical College, Addis Ababa, Ethiopia; ^3^Primary Immune Deficiency Clinic Laboratory of Clinical Immunology and Microbiology NIAID, NIH, North Bethesda, MD, USA; ^4^Dermatovenerologist, Pediatric Dermatology Fellow, Addis Ababa University College of Health Sciences, Addis Ababa, Ethiopia; ^5^Dermatovenerologist, Photoallergologist, Addis Ababa University College of Health Sciences, Addis Ababa, Ethiopia; ^6^Dermatovenerologist, Dermatopathologist, Addis Ababa University College of Health Sciences, Addis Ababa, Ethiopia; ^7^Dermatovenerologist, Debre Birhan Specialized Hospital, Debre Birhan, Ethiopia; ^8^Dermatovenerologist, Dermatosurgeon, Addis Ababa University College of Health Sciences, Addis Ababa, Ethiopia; ^9^Pediatrics Resident, Department of Pediatrics and Child Health, St.Paul's Hospital Millennium Medical College, Addis Ababa, Ethiopia; ^10^Division of Pulmonary and Critical Care, Department of Pediatrics and Child Health, St.Paul's Hospital Millennium Medical College, Addis Ababa, Ethiopia

## Abstract

**Background:**

Mendelian susceptibility to mycobacterial diseases (MSMD) is an inborn error of immunity categorized as defects in intrinsic and innate immunity. MSMD is characterized by vulnerability to less virulent mycobacteria, such as *Bacillus* Calmette-Guérin (BCG) vaccine strains, as well as environmental mycobacteria (EM). The definitive diagnosis is made by genetic analysis. Treatments constitute antimycobacterial, interferon-gamma, surgery, and hematopoietic stem cell transplantation (HSCT), which is the only known curative treatment. The mortality rate ranges from 40% to 80% depending on the severity of the mutation.

**Case:**

A 13-year-old female patient had multiple hospital visits since the age of 6 months. The most striking diagnosis was repeated mycobacterial infections. She had *tuberculosis* affecting lymph nodes, skin and soft tissue, bone and joints, the lungs, and epidural and paraspinal regions. She has taken all the childhood vaccines, including BCG. She has been treated four times with first-line and once with second-line antituberculosis drugs. Currently, she is on treatment for nontuberculous mycobacteria and is receiving interferon-gamma. Genetic studies showed autosomal dominant Mendelian susceptibility to mycobacterial disease due to IFNG-R1 defect.

**Conclusion:**

To the authors' knowledge, this is the first case report of Mendelian susceptibility to mycobacterial diseases secondary to interferon gamma receptor 1(IFNG-R1) defect in Ethiopia. Although it has been immensely challenging, our multidisciplinary team has learned a lot from the clinical presentation, diagnosis, and management of this child.

## 1. Introduction

The Mendelian susceptibility to mycobacterial diseases (MSMD) is an inborn error of immunity classified in the category of defects of intrinsic and innate immunity as per the 2022 updated classification from the international union of immunological society expert committee [1]. This defect has been observed particularly in a cohort of infants who have received the BCG vaccine at birth and who later developed disseminated BCG infection, and the clinical syndrome has been termed Mendelian susceptibility to mycobacterial diseases (MSMD) [2]. The first genetic etiology of MSMD was discovered in 1996 and Interferon-*γ* receptor-1 (IFN*γ*R1) deficiency was the first to be identified among the other causes of MSMD [3].

There are three types of inheritance patterns of MSMD :  autosomal recessive (AR), autosomal dominant (AD), and X-linked recessive (XR). These three patterns, as well as their subtypes, differ in the functional impairment based on residual response to stimulation of receptors, with complete deficiency and partial deficiency [4].

In this clinical case presentation, we are interested in the cytokine Interferon-*γ* as well as its receptors on macrophages. The T-lymphocytes work hand in hand with phagocytes; they recognize a unique antigen via T-cell receptors when it is presented to them by antigen-presenting cells (APCs). These APCs are macrophages, dendritic cells, and B-cells, and they express major histocompatibility complex–type-II (MHC II) molecules [5]. The two key subsets of T-cells are CD4 and CD8 T-cells. The CD4 T-cells are called T-helper cells, and are further divided into T-helper-1(Th1), T-helper-2 (Th2), and T-helper-17(Th-17) cells [6, 7].

T-helper-1 cells' major functions are to produce cytokines, activate other cells, and direct immune responses. These cytokines are interleukin 2 (IL2) and interferon-gamma (IFN-*γ*) [3]. Interferon-gamma is also produced by natural killer (NK) cells [8]. In the process of phagocytosis, when macrophages swallow up microorganisms, they produce IL-12. Intracellular signaling through IL-12R is mediated by STAT-4 phosphorylation, which activates IFN *γ* production by T-helper-1 cells. IFN *γ* in return stimulates macrophages to mediate the intracellular killing of microorganisms. IFN *γ* acts on IFN *γ* receptors in phagocytes and is composed of IFN *γ*R1 and IFN *γ*R2.3.

In patients with disorders of IFN *γ* and their receptors, their macrophages fail to kill intracellular organisms such as mycobacteria, *Salmonella Typhimurium*, Listeria, *Klebsiella*, *Candida*, and intercellular parasites such as Leishmania [9–16]. This case report is tuned around a girl with an IFN *γ*R1 defect.

## 2. Case Presentation

We present a 13-year-old girl with repeated infections since early infancy; only prominent conditions throughout her course of illness are reported here.

Her first visit was when she was 45-days-old. She had an exaggerated wound on the BCG vaccine site (right deltoid area) . She was treated with antibiotics and wound care and improved.

At 14 months old, she was diagnosed with tuberculosis (TB) lymphadenitis, evidenced by bilateral enlarged cervical nodes with constitutional symptoms of 1-month duration. Her ESR was 130 mm/hr and fine needle aspiration (FNA) from her lymph nodes revealed caseous necrosis. Her HIV test was negative; she was treated with first-line anti-TB drugs (2RHZE/4RH).

When she was 3 years old, she presented with a cough and fever for 3 weeks. Upon physical examination, she had generalized lymphadenopathy and left posterior lung field crepitation. Laboratory investigation showed leukocytosis with left shift and raised ESR. Her chest X-ray revealed left paratracheal and basal ill-defined opacity. Tests for malaria, hepatitis B, and hepatitis C were all negative. Gene expert MTB/RIF test and acid-fast staining from gastric aspirate did not show *Mycobacterium Tuberculosis*, FNA from lymph nodes showed caseous necrosis, and she was treated with first-line anti-TB (2RHZE/4RH) for the second time.

At the age of 4 years and 3 months, she presented with right lower extremity pain, limping, and swelling over her head. On physical examination, she had boggy swelling over her scalp, multiple enlarged cervical lymph nodes, and anterior chest wall swelling at two sites. Her ESR was 124 mm/hr. Her abdominal ultrasound showed hepato-splenomegaly and multiple enlarged iliac groups of lymph nodes bilaterally. Fine needle aspiration from the chest wall abscess and lymph nodes suggested features of *tuberculosis*. Multi-drug-resistant TB (MDR TB) was considered clinically, but it was not possible to confirm with TB culture and drug sensitivity tests. She was treated with second-line anti-TB drugs for 2 years. This was the time that her mom claimed, she had marked improvement.

**Figure 1:**
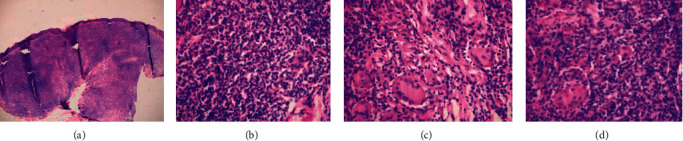
(a) H and E stained wedge shaped incisional biopsy, bisected from the skin on her trunk. (b) Diffused granulomatous with multiple histiocytes with a background of lymphocytes periadnexal inflammatory cell infiltrate. (c) and (d) Multinucleated giant cell and Touton giant cells. All were suggestive of lupus vulgaris. No acid-fast bacilli were seen.

At the age of 7 years and 9 months, she presented with right-side limping, loss of appetite, weight loss, fever, and occasional cough. On physical examination, she had boggy swellings over her scalp in three different areas; acne-like rashes on the back, trunks, and arms; and shortening of the right lower limb with limited range of movement. Her MRI showed lumbosacral multifocal osteomyelitis; unilateral sacroiliac arthritis with adjacent epidural abscess; and lytic lesions over the left femoral head and neck. The skull X-ray was normal. Her C-reactive protein (CRP) and parathyroid hormone were within normal limits. Rheumatoid factor (RF) and antinuclear antibody (ANA) were both negative. The FNAC from the scalp lesion showed caseous granulomatous inflammation and the biopsy from the skin lesion was suggestive of lupus vulgaris. (Figures 1(a)–1(d)). The disseminated mycobacterial disease was considered, and she was treated with anti-TB for the 4th time (2RHZE/10RH).

**Figure 2:**
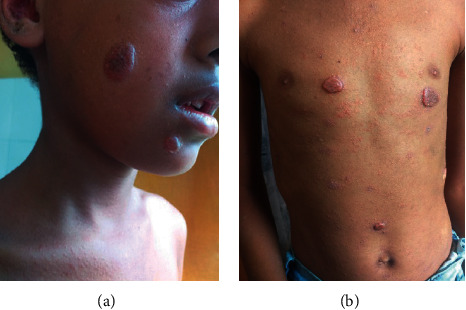
a and b well-demarcated skin-colored indurated plaque lesion with the fine-scale on the right side of cheek, chin, chest, and abdomen.

**Figure 3:**
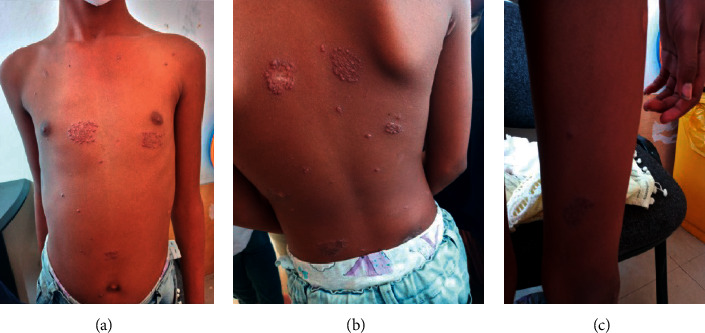
a,b, and c monomorphic grouped skin-colored papules and sparse discrete skin-colored papules on the chest, back, and left thigh area.

**Figure 4:**
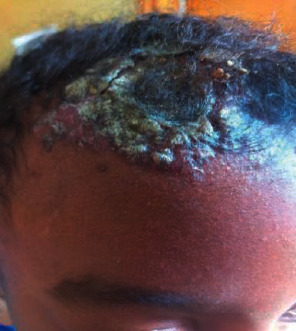
Yellowish-crusted plaque with indurated erythematous background on the right side of the anterior scalp and forehead, picture was taken around 9 years of age.

Despite the treatment with the anti-TB, the indurated plaque skin lesions (Figures 2(a) and 2(b)) became cauliform-like (Figures 3(a)–3(c)) and the boggy scalp lesion became crusted (Figure 4(a)); another skin biopsy was suggestive of juvenile xanthogranuloma. Other blood tests and ophthalmologic evaluation were normal, and she completed her anti-TB course.

**Figure 5:**
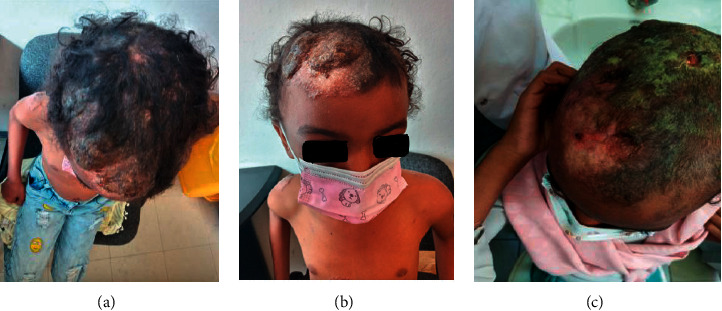
(a),(b) Diffuse yellowish-crusted plaque with associated hair loss on the anterior scalp area at 11 years and 3 months (5c) healing scalp lesion after antibiotic and anti TB treatment at the age of 11 years and 6 months.

At the age of 11 yr and 3 months, she developed right flank pain with swelling, associated with low-grade fever, in addition to the existing scalp and skin lesions. Her vital signs were stable, she had abdominal tenderness and a limited range of movement of the right hip joint. She had a yellowish-crusted plaque with an indurated erythematous background on the right side of the anterior scalp and forehead (Figures 5(a)–5(c)), and a well-demarcated skin-colored indurated plaque with a fine scale on the right side of the cheek, chin, chest, and abdomen. Her abdominal ultrasound and MRI were suggestive of right-side unilateral sacroiliac arthritis with adjacent paraspinal abscess; her ESR was raised with leukocytosis and anemia. FNAC from lymph nodes showed a granulomatous lesion with caseous necrosis; blood PCR showed the *Mycobacterium* Tuberculosis complex (species identification was not possible). Anti-TB (2RHZE/10RH) was started for the 5^th^ time.

## 3. Most Recent Management and Course

At the age of 12 years and 10 months, she came back with a complaint of cough, high-grade fever, and chest pain of 3 weeks' duration. She was chronically sick-looking, tachypneic with an oxygen saturation of 56% (corrected with 3 liters of oxygen). She was severely underweight and stunted, and her body mass index (BMI) was <5th percentile (CDC BMI chart for her age).

**Figure 6:**
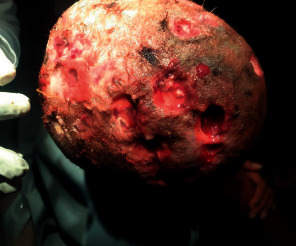
Multiple punched-out scalp lesions and pictures were taken at 12 years and 10 months right after the debridement.

She had frontal bossing, multiple deep punched-out ulcers over the scalp covering an approximate area of 15 by 12 centimeters on the frontal, temporal, and parietal parts of the head (Figure 6).

She had proptosis of eyes, multiple nontender enlarged lymph nodes (2–4 cms) at the axillary and inguinal regions, coarse crepitation over the left upper third of lung field, hepatomegaly of 16.5 cms, and spleen size of 2 cms. She had a nodular ulcer 1 cm by 1 cm over the two shoulders. She had multiple hyper and hypopigmented circular scars. She had a deep scar over her left buttock. She was extremely irritable; slight deviation of the left eye to the left side; other neurologic examinations were normal.

**Figure 7:**
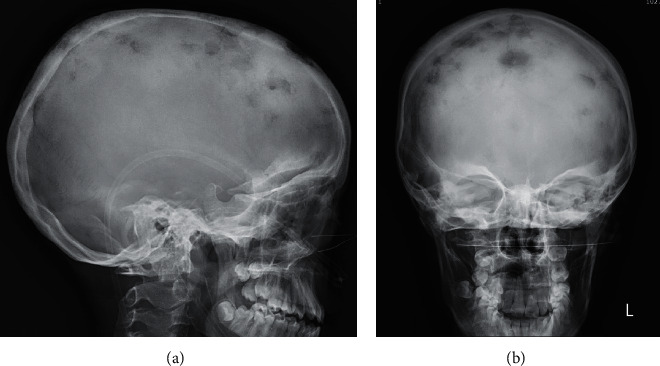
(a) and (b) Skull X-ray of the 13 years old girl with MSMD from Ethiopia: multiple trans-diploic lesions with inner and outer calvarial bone destruction; findings were suggestive of delayed/arrested sphenoid sinus pneumatization.

Her investigations had a WBC of 23800/mm3, with a neutrophil count of 83%; her ESR was 140 mm/hr; hemoglobin was 9.2 gm/dl; Gene-Xpert from sputum showed no mycobacteria *tuberculosis*. Thyroid, liver, and renal function tests were within normal limits. Her Skull X-ray showed multiple punched-out lytic lesions without sclerotic border involving frontal, parietal, and occipital bones bilaterally (Figures 7(a) and 7(b)).

Ultrasound of the abdomen showed a liver size of 15.8 cm, and a spleen size of 12 cm. Both had smooth contours and uniform echo textures.

### 3.1. Her Chest CT (Video Clip: 1) Revealed

Multiple lytic bone lesions involving the bilateral humeral head, both right and left acromion processes of scapula and body of scapula, right 2^nd^ to 6^th^ rib, left 3^rd^ to 5^th^ rib with a narrow zone of transition and no associated periosteal reaction or sclerosis except for mild physical sclerosis at the left humeral head, with C6 vertebral compression fracture, heterogeneously enhancing anterior mediastinal mass with punctate calcification likely to be an enlarged thymus with calcification, bilateral nonsegmental multifocal consolidative airspace opacities, bilateral pleural effusion (right side-scant, left side-moderate), bilateral axillary and cervical lymphadenopathy.

### 3.2. Her Brain MRI (Video Clip-2) Revealed

Multiple trans-diploic lesions with inner and outer calvarial bone destruction involving frontal-parietal and occipital regions with subtle parenchymal edema from the mass effect from frontal lesion suggestive findings of delayed/arrested sphenoid sinus pneumatization.

Genetics test: Sequencing analysis and deletion/duplication testing of the 407 genes showed a pathogenic variant in *IFNGR1,* (Med Gen UID: 863300) c.819_822del (p.Asn274Hisfs*∗*2) suggestive of IFN-*γ*-R1 defect and this result is consistent with a predisposition to autosomal dominant mendelian susceptibility to mycobacterial disease (MSMD) one increased risk allele, *NOD2* (MedGen UID: 348835) c.2722 G > *C* (p.Gly908Arg) was identified, which could be associated with an increased risk of Crohn's disease.

She was managed with intravenous ceftazidime and vancomycin, followed by Meropenem for 2 weeks. Rifampicin, INH, ethambutol, azithromycin, and Cotrimoxazole were given to cover for possible nontuberculous mycobacteria and superinfection with *staphylococcus aureus*, *Salmonella*, and Listeria. Surgical debridement was done and pathology from the necrotic scalp and lytic skull bone fragments was suggestive of only chronic inflammation (not LCH).

Thereafter, she went to the National Institute of Allergy and Infectious Disease (NIAID) in Maryland where she received a PCR test from sputum and scalp wound which showed *Mycobacterium avium* complex (MAC) and culture grew *Mycobacterium Intracellulare*; and a PET scan to see the extent of disease showed spots of osteomyelitis on arms, shoulders, legs, ribs, the skull, and sacral area. She was started on Azithromycin, Linezolid, Moxifloxacin, and Bedaquiline. The ophthalmologist's evaluation revealed uveitis; she was started on steroid eye drops and her vision has improved.

**Figure 8:**
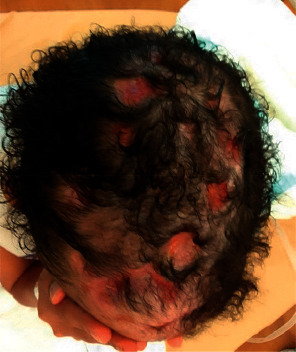
Healing scalp lesions. The picture was taken just recently while being treated at NIAID.

After three months of the above therapy, she developed prolonged QT syndrome as a side effect of bedaquiline, and it was discontinued. The sensitivity pattern of the identified MAC from the sputum and mediastinal mass was resistant to azithromycin, however the MAC from the scalp was sensitive to azithromycin, hence azithromycin was continued and clofazimine was added. Surgery to excise the mediastinal mass differed. interferon gamma was started. Now she is not requiring oxygen, she has gained weight and the scalp wound is healing (Figure 8), even though her progress is not satisfactory Hence, bone marrow (BM) transplant is planned and her donors are being investigated for matching.

### 3.3. Additional Medical History

Her parents are in good health and have no consanguinity. She was born at term with an uneventful neonatal period, with no delay in the falling of the umbilical stump. She has a nine-year-old sister and a five-year-old brother, both in good health condition. She was a fourth-grade student with average performance but has now discontinued school because of her illness. There is no known family history of a similar chronic illness.

## 4. Discussion

The diagnosis of primary immune deficiency in settings with limited resources, such as ours, is always challenging due to the lack of proper diagnostic modalities. When this girl came suffering from repeated infections despite normal to high WBC and normal platelet counts, the most important diagnosis considered was functional phagocytosis defect. Children with phagocytic defects present with deep tissue infections, pneumonia, adenitis, or osteomyelitis rather than bloodstream infections similar to our patient. The initial phagocyte functional disorders considered were chronic granulomatous disease (CGD) and leukocyte adhesion defect-1(LAD-1), which both have been ruled out after the genetics study [17].

Langerhans cell histiocytosis (LCH) was another differential considered due to the lytic bone lesions, and thymic and skin involvement she had. The most common bone involved in LCH is the skull with a punched-out lesion with or without a sclerotic border. Additionally, long bones, vertebrae, and the mandible can be affected. Raised skin lesions with red-brown crusted areas, bumps, and ulceration of the scalp, collapsed lungs, hematopoietic system, and CNS can also be affected similar to our patient's findings. However, repeated bone marrow and pathologic examinations from the necrotic scalp and lytic skull bone fragments were not suggestive of LCH [18].

Our patient's genetic analysis revealed an autosomal dominant mendelian susceptibility to mycobacterial disease due to IFN-*γ*-R1 defect [*IFNγR1* (MedGen UID: 863300) c.819_822del (p.Asn274Hisfs*∗*2)]. Autosomal dominant partial IFN*γ*R1 deficiency is characterized by increased expression of receptors on the cell surface due to a defect in the recycling of IFN-*γ*-R1 receptors. These types of disorders are associated with defects in interferon *γ* (IFN-*γ*) production or response to IFN-*γ* results in macrophages' failure to kill intracellular organisms such as Mycobacteria. In particular patients with MSMD are at risk for osteomyelitis caused by mycobacteria, as was observed in our patient. [17–20].

Our patient received the BCG vaccine at birth, which is one of the routine childhood vaccines given in our setting. At 45 days old, she had exaggerated ulceration at the site of vaccination, which eventually healed with wound care and antibiotics. However, we also assume that the mycobacterial infections in our patient, which occurred at 1 year and 2 months of age and 3 years of age, could also be attributed to the disseminated BCG-osis. In two Iranian studies, 60% of patients with regional BCG-itis or disseminated BCG-osis presented within one year of vaccination; in some, the onset was delayed as long as 72 months after the vaccine [21]. In an Indian cohort disseminated BCG-osis was the commonest presenting disease in 82% of patients with MSMD, with a median age of presentation of 6 months [22].

The underlining cause of disseminated forms of BCG infection was MSMD in 69.5% out of the 47 pediatric patients suspected in the Iranian studies [21].

Other intracellular bacteria such as *Salmonella Typhimurium*, Listeria, and *Klebsiella* are possible organisms to cause disseminated infections including chronic osteomyelitis. Though blood cultures from our patient did not reveal these organisms, the chronicity of her illness and her poor response to short-term antibiotics treatment and anti-TB drugs made us think that these bacterial infections could have been possibilities, and we have empirically treated her with co-trimoxazole- Sulfamethoxazole.

Patients with MSMD are at risk for repeated *Mycobacterium tuberculosis* infections. In the Colombian cohort, 56.5% (13) of 23 patients had *tuberculosis* [23] Our patient has been repeatedly diagnosed clinically with disseminated *tuberculosis* to the lungs, liver, spleen, lymph nodes, bones, and skin. The histopathologic investigations of the lymph nodes and skins were repeatedly suggestive of caseous necrosis, and PCR once was suggestive of *Mycobacterium tuberculosis* complex. We believe that the clinical decisions to treat her with repeated first-line regimens were sound judgments made by our clinicians as Ethiopia was identified as among the first 20 high TB burden countries with a TB incidence rate of 132/100,000 population as per the 2020 world bank data [24].

Repeated *tuberculosis* usually alarms clinicians to consider either HIV infection or multidrug-resistant *tuberculosis* (MDR); her HIV test was negative, hence she was treated with a second-line anti-TB regimen at the age of 4 years.

The consideration of primary immune deficiency states is often delayed because of the unavailability of the tests and the limited knowledge of primary physicians.

Despite anti-TB therapy, our patient continued to have a lingering infection throughout the years with minimal and temporary response to anti-TB drugs. In such scenarios it is worth considering nontuberculous mycobacteria as an alternative or additional diagnosis, as was observed in the Colombian MSMD cohort, in which 3 (13%) of the 23 patients had Environmental Mycobacteria (EM) infections.

Most recently, at NIAID, PCR and culture from sputum and scalp wounds identified *Mycobacterium Intracellular*, and specimens from the lungs and scalps showed variable sensitivity to azithromycin. She is now receiving azithromycin, linezolid, moxifloxacin, bedaquiline, clofazimine, and interferon gamma. She has weight gain with unsatisfactory wound healing. Therefore, hematopoietic stem cell transplantation is planned.

The treatment modalities of patients with MSMD include antibiotics, antimycobacterial IFN *γ* therapy, surgery, and hematopoietic stem cell transplantation (HSCT), of which the latter is the only known curative treatment. The mortality rate ranges from 40% to 80% depending on the severity of the mutation.

## 5. Conclusion

In conclusion, our patient exhibited regional BCG-itis and disseminated BCG-osis, *Mycobacterium tuberculosis,* and Environmental *Mycobacterium* (nontuberculous mycobacteria) infections. Although it was difficult to make specific identification, and targeted treatment on each of the occasions while she was in Ethiopia, clinician decisions were made carefully based on the available molecular, serologic, pathologic, histological, and imaging studies. She was treated with both repeated first-line and second-line anti-tuberculosis regimens. Additionally, before her travels, she was covered with regimens to treat possible EM and other intracellular bacteria.

To the authors' knowledge, this is the first case report of Mendelian susceptibility to mycobacterial diseases secondary to interferon gamma receptor 1(IFNG-R1) defect from Ethiopia. Although it has been immensely challenging, our multidisciplinary team has learned a lot from the clinical presentation, diagnosis, and management of this child, and we are presenting this case so that other clinicians may learn from it.

## Data Availability

The data that support the findings of this study are available from the corresponding author upon reasonable request.
